# Draft Genome Sequences of Two *Enterobacter cloacae* subsp. *cloacae* Strains Isolated from Australian Hematology Patients with Bacteremia

**DOI:** 10.1128/genomeA.00756-17

**Published:** 2017-08-17

**Authors:** Lex E. X. Leong, David Shaw, Lito Papanicolas, Diana Lagana, Ivan Bastian, Geraint B. Rogers

**Affiliations:** a Infection and Immunity, South Australian Health and Medical Research Institute, Adelaide, South Australia, Australia; b College of Medicine and Public Health, Flinders University, Bedford Park, South Australia, Australia; c Infectious Diseases Department, Royal Adelaide Hospital, Adelaide, South Australia, Australia; d SA Pathology, Adelaide, South Australia, Australia

## Abstract

*Enterobacter cloacae* is a common member of the gut microbiota in healthy individuals. However, it is also an opportunistic pathogen, capable of causing bacteremia. We report the draft genomes of two *Enterobacter cloacae* subspecies *cloacae* strains isolated from hematology patients with bacteremia. Both isolates carry genes encoding extended-spectrum β-lactamases.

## GENOME ANNOUNCEMENT

Bacteria of the *Enterobacter cloacae* complex are commensal enteric bacteria of the human gastrointestinal tract. In recent years, these bacteria have taken on clinical significance as opportunistic pathogens that cause severe bacteremia through endogenous translocation from the host gut (1–3). *E. cloacae* and other *Enterobacter* spp. have intrinsic and inducible resistance against broad-spectrum cephalosporins through chromosomal AmpC β-lactamase (1). *Enterobacter* strains are also capable of acquiring plasmids that encode multiple drug resistance mechanisms, such as extended-spectrum β-lactamases (ESBL) that hydrolyze a broader range of cephalosporins, including fourth-generation cephalosporins (cefepime) (4–7). Infections by these multidrug-resistant strains pose a significant therapeutic challenge.

We report the draft genome sequences and antibiotic resistance gene profiles of two *Enterobacter cloacae* subsp. *cloacae* isolates (49530189 and 60830776) from the *Enterobacter cloacae* complex, which were isolated from two different individuals with hospital-acquired bacteremia. Both isolates were obtained by blood culture, and their antibiotic susceptibilities were determined using EUCAST breakpoints with the BD-Pheonix automated platform (Becton, Dickinson, NJ, USA) and double-disc testing. Isolate 49530189 was determined to be resistant to ciprofloxacin, co-trimoxazole, cefepime, and other β-lactams. Isolate 60830776 differed slightly, being intermediately resistant to cefepime and resistant to ciprofloxacin, co-trimoxazole, aztreonam, gentamicin, and tobramycin.

Genomic DNA was isolated using an EZ-10 Spin genomic DNA kit (Bio Basic Canada, Inc., Ontario, Canada). Whole-genome amplicon libraries were prepared using an Illumina Nextera XT DNA sample preparation kit (Illumina, Inc., CA) and sequenced on an Illumina NextSeq platform with a NextSeq 500/550 mid-output kit (v2 Illumina) (2 × 150-bp cycles).

A total of 3,066,602, and 1,876,696 paired-end reads for isolates 49530189 and 60830776, respectively, were mapped to the *Enterobacter cloacae* subsp. *cloacae* ATCC 13047 reference genome (GenBank accession no. NC_014121), yielding average coverages of 78-fold and 49-fold, respectively. Downstream processing was performed using the Nullarbor pipeline (see https://github.com/tseemann/nullarbor). The draft genome of isolate 49530189 consisted of 170 contigs, with a total of 5,467,426 bp and G+C content of 54.8%. The draft genome of isolate 60830776 consisted of 140 contigs, with a total of 5,168,633 bp and G+C content of 54.6%. Isolates 49530189 and 60830776 have 5,239, and 4,879 coding domain sequences, respectively. Both draft genomes have six sets of rRNA genes. The core genome between the 2 isolates consists of 2,332 genes.

While the two isolates have differing profiles of antibiotic resistance genes, both carry genes that confer resistance to aminoglycosides, β-lactams, fosfomycin, olaquindox, quinolone, sulfonamide, and tetracycline. However, isolate 60830776 also harbors resistance genes toward chloramphenicol (*catB3*), and macrolide (*mphA*). The two isolates are AmpC negatve and ESBL positive. Isolate 49530189 carries AmpC (*bla*_ACT-9_) and CTX-M (*bla*_CTX-M-9_) β-lactamases, while isolate 60830776 harbors AmpC (*bla*_ACT-16_), TEM-1 (*bla*_TEM-1B_), SHV (*bla*_SHV−12_), and OXA-1 (*bla*_OXA-1_) β-lactamases. These *E. cloacae* draft genomes are a useful resource that enables us to better understand antibiotic resistance mechanisms, phenotype-genotype correlations, and evolutionary history.

### Accession number(s).

These whole-genome shotgun projects have been deposited in DDBJ/ENA/GenBank under the accession numbers NJCZ00000000 (49530189) and NJDA00000000 (60830776). The versions described here are the first versions.
